# A Rare Case of Inflammatory Cardiac Mass in the Young: Diagnostic Uncertainty and Therapeutic Challenges

**DOI:** 10.7759/cureus.72791

**Published:** 2024-10-31

**Authors:** Zubair Farooq, Preeti Gupta, Abhinav Aggarwal, Sachin Gautam

**Affiliations:** 1 Cardiology, Vardhman Mahavir Medical College and Safdarjung Hospital, New Delhi, IND

**Keywords:** cardiac masses, cardiac tumors, case report, right atrial mass, steroids, tricuspid valve

## Abstract

Inflammatory cardiac masses are rare and often mimic neoplastic or thrombotic lesions, posing significant diagnostic challenges. These masses are typically identified incidentally during imaging studies, such as echocardiography, and further evaluation with cardiac magnetic resonance imaging (MRI) is required for accurate tissue characterization. Early recognition and appropriate management are crucial to prevent complications.

We present the case of a 20-year-old previously healthy female who presented with episodic palpitations lasting seconds to minutes over a two-month period. Transthoracic echocardiography revealed a hyperechoic mass attached to the tricuspid valve, and cardiac MRI indicated inflammatory involvement of the myocardium, with myocardial edema and hyper-trabeculation. Laboratory investigations ruled out infectious etiologies, and she was started on corticosteroid therapy (prednisolone 30 mg daily, tapered over four weeks) along with metoprolol.

Despite a reduction in symptoms with treatment, follow-up echocardiography showed persistence of the cardiac mass, raising questions about the efficacy of corticosteroid therapy. During this period, the patient also experienced an episode of ventricular premature contractions (VPCs), detected through smartwatch tracing, although these were not detected on a 24-hour Holter monitor. No significant mass reduction was observed, and her condition remained stable on beta-blocker therapy.

This case highlights the diagnostic and therapeutic complexities of inflammatory cardiac masses in young patients. The persistence of the mass despite corticosteroid treatment suggests the potential need for alternative interventions, such as surgical evaluation or biopsy, to confirm the etiology and guide further management. Inflammatory cardiac masses can closely mimic neoplastic growths, necessitating a multidisciplinary approach to optimize outcomes and prevent misdiagnosis. Early detection, accurate diagnosis through advanced imaging, and close follow-up are essential to guide appropriate treatment and prevent complications.

## Introduction

Cardiac masses, though uncommon, present a diagnostic challenge due to the wide range of potential etiologies, including benign and malignant tumors, thrombi, and inflammatory masses [1]. Inflammatory cardiac masses are particularly rare, with limited data on their prevalence, especially in young, otherwise healthy individuals. These masses can closely mimic more common cardiac conditions such as neoplasms or thrombotic lesions, making timely and accurate diagnosis critical [2]. Their potential to cause significant morbidity through arrhythmias, obstruction, or embolic events further underscores the importance of prompt intervention.

Inflammatory cardiac masses can arise in the context of infectious diseases, autoimmune conditions, or idiopathic inflammation. Tuberculomas, while exceedingly rare, serve as an example of an inflammatory mass that may present without a clear history of tuberculosis, complicating diagnosis [3]. Other inflammatory conditions, such as sarcoidosis, myocarditis, and autoimmune diseases like lupus and rheumatoid arthritis, can similarly lead to the formation of masses through immune cell accumulation, scarring, or granulomatous inflammation.

This case report describes a 20-year-old female with an inflammatory cardiac mass presenting at the tricuspid valve, a finding that is both rare and diagnostically challenging. The case is noteworthy due to the persistence of the mass despite corticosteroid therapy and its atypical presentation in a young patient without a significant history of autoimmune or infectious disease. It highlights the difficulty in managing inflammatory cardiac masses, the need for a multidisciplinary approach, and the potential role of advanced imaging techniques in guiding treatment decisions [4,5]. This report adds to the limited body of literature on inflammatory cardiac masses and emphasizes the necessity of careful diagnostic evaluation to distinguish these from other intracardiac lesions.

## Case presentation

**Figure 1:**
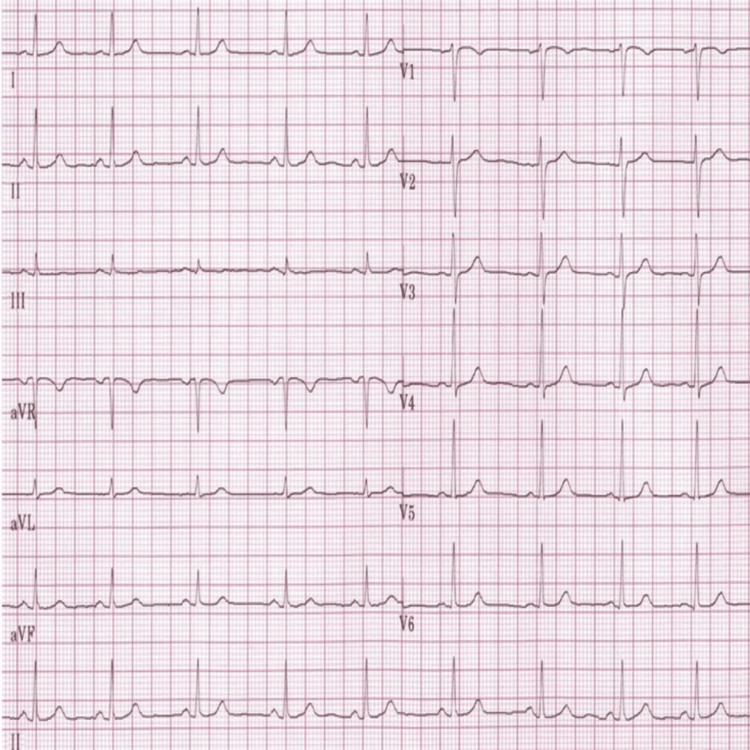
12-lead ECG showing normal sinus rhythm with an axis around +60 and no evidence of pre-excitation, recorded in standardization (1 mV = 10 mm) and a standard sweep speed of 25 mm/s.

A 20-year-old previously healthy female with no co-morbidities presented with complaints of episodic palpitations for the past two months. The episodes lasted for a few seconds to minutes, were regular in nature, and occurred two or three times a week. There were no other symptoms suggestive of cardiovascular involvement like chest pain, dyspnea or fatigue, etc. Her physical examination, including cardiovascular assessment, was unremarkable. Baseline ECG shown in Figure 1 showed sinus rhythm with an axis around 60 degrees and no evidence of pre-excitation.

**Figure 2:**
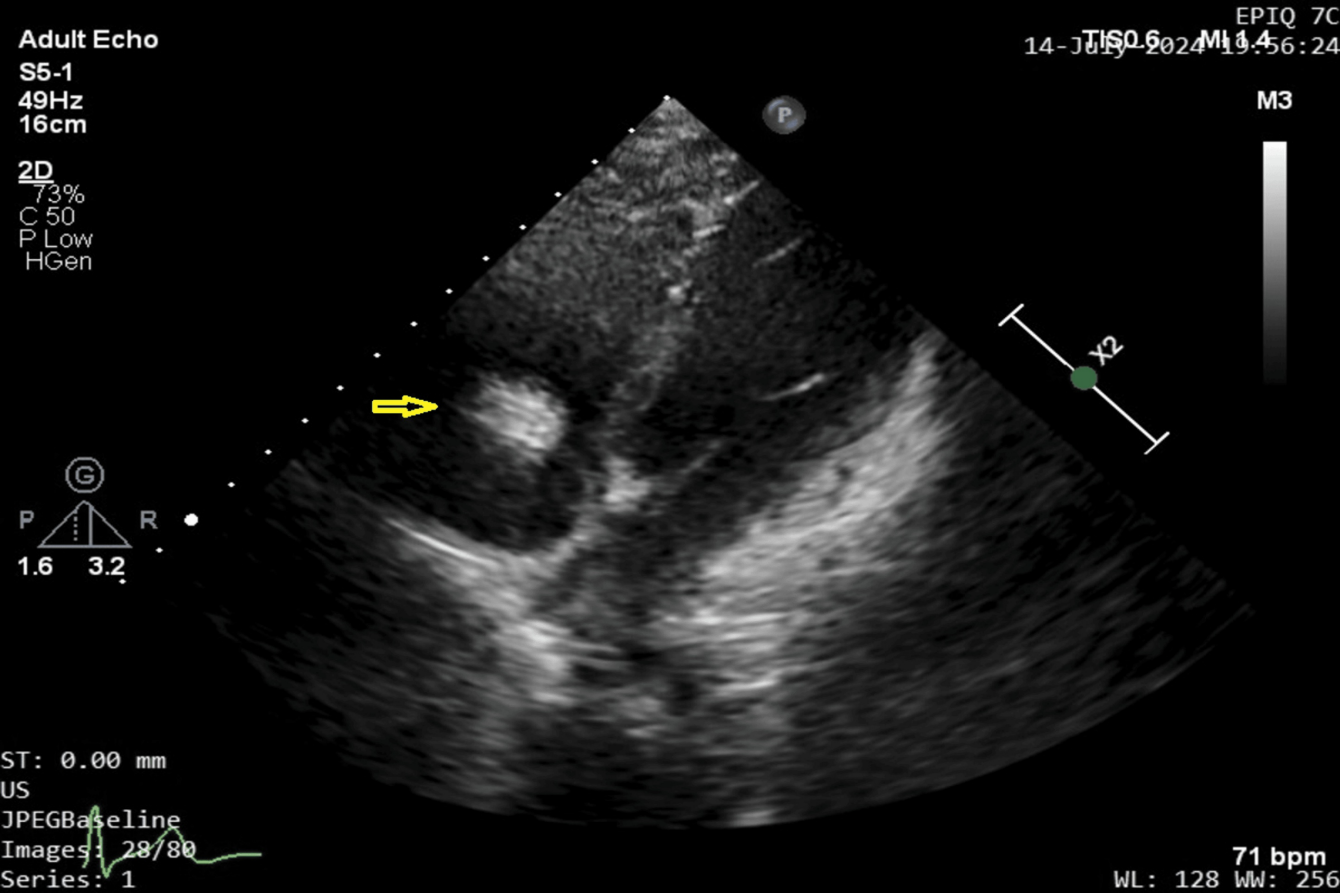
Modified 4-chamber view on echocardiography suggestive of a hyperechoic mass (yellow arrow) measuring 4 x 6 mm attached to right atrial aspect of tricuspid leaflet.

Transthoracic echocardiography revealed a hyperechoic mass attached to the right atrial aspect of the tricuspid valve as shown on the modified 4-chamber view in Figure 2.

**Figure 3:**
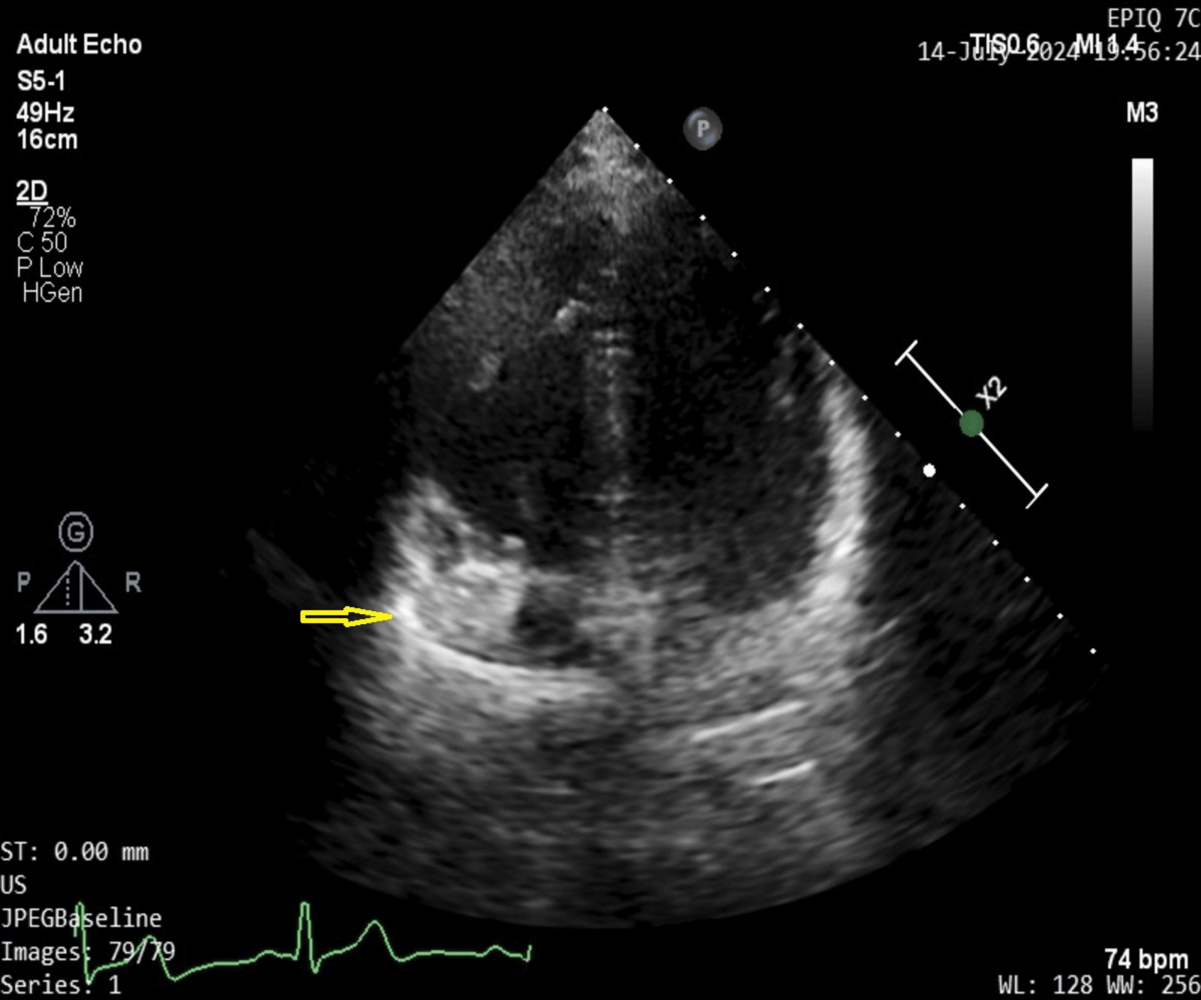
4-chamber view on transthoracic echocardiography showing hyperechoic mass (yellow arrow) in the right atrium.

The mass can be visualized in apical 4-chamber view as seen in Figure 3.

**Figure 4:**
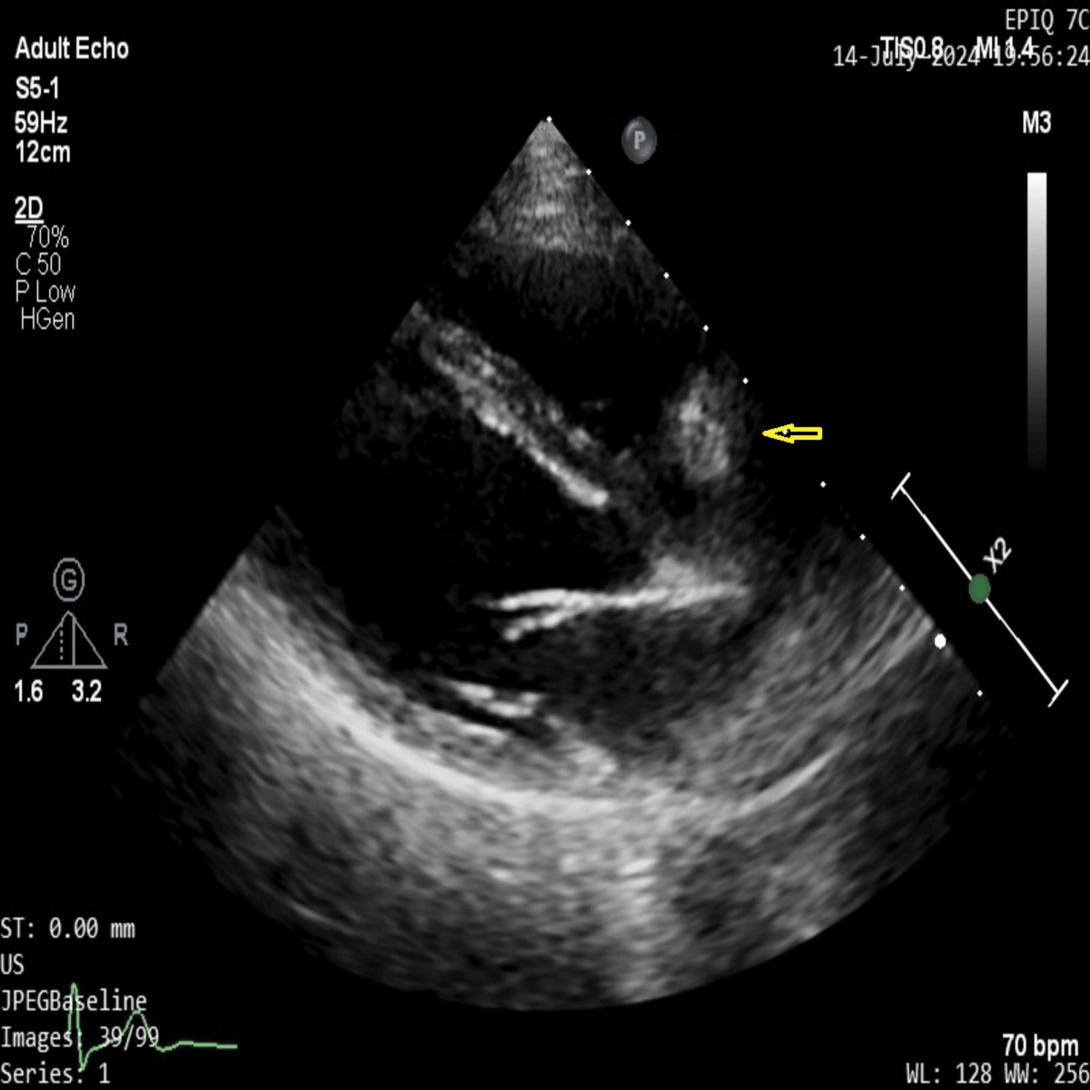
RV inflow view on transthoracic echo showing location of the hyperechoic mass (yellow arrow) in relation to tricuspid valve.

The mass was also well localized in RV inflow view as shown in Figure 4.

**Figure 5:**
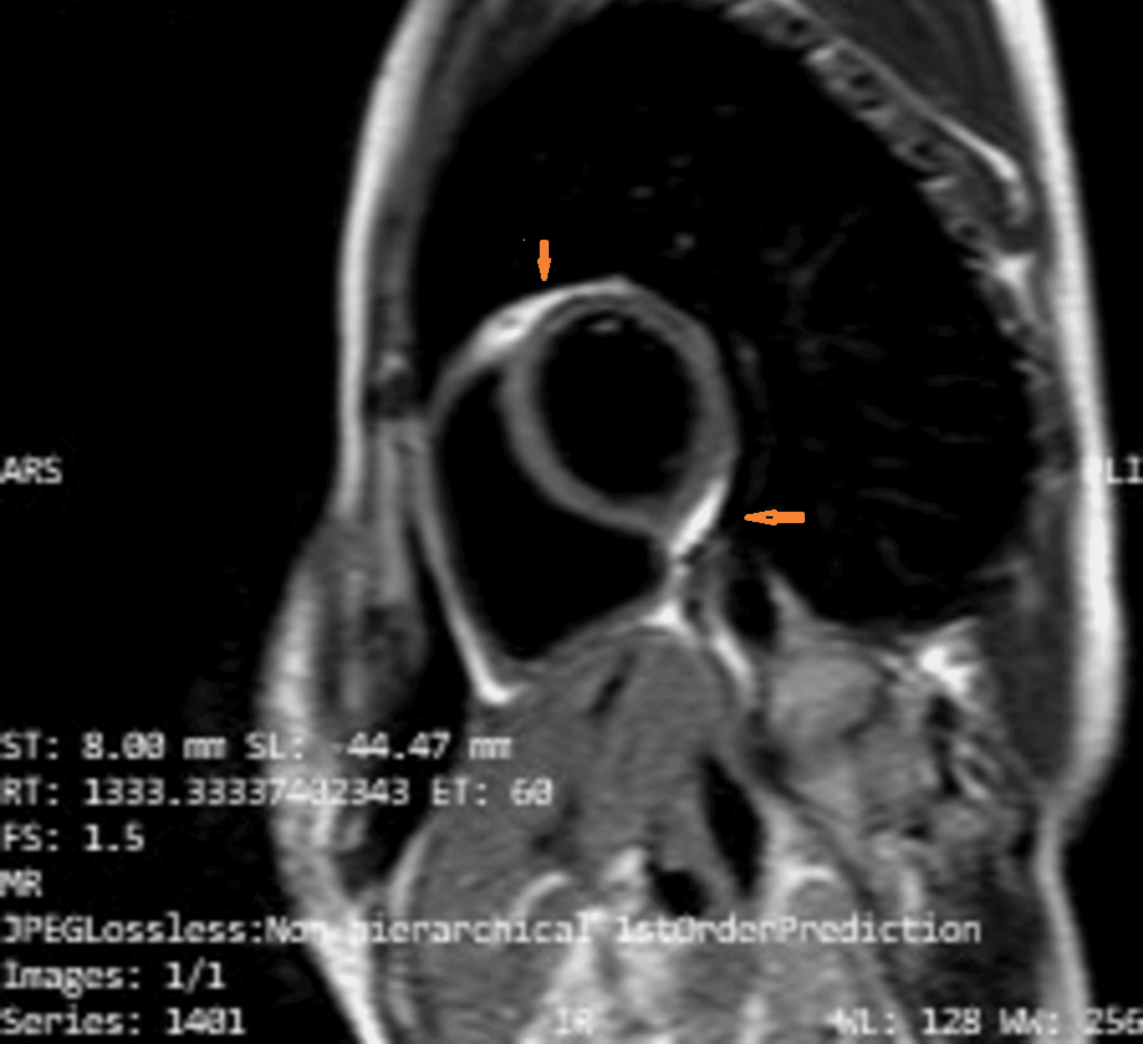
T2-weighted Turbo Spin Echo with Fat Saturation MRI sequence showing involvement of the left ventricular myocardium with basal and mid-cavity antero-inferior myocardial hyperintensity (orange arrows).

**Figure 6:**
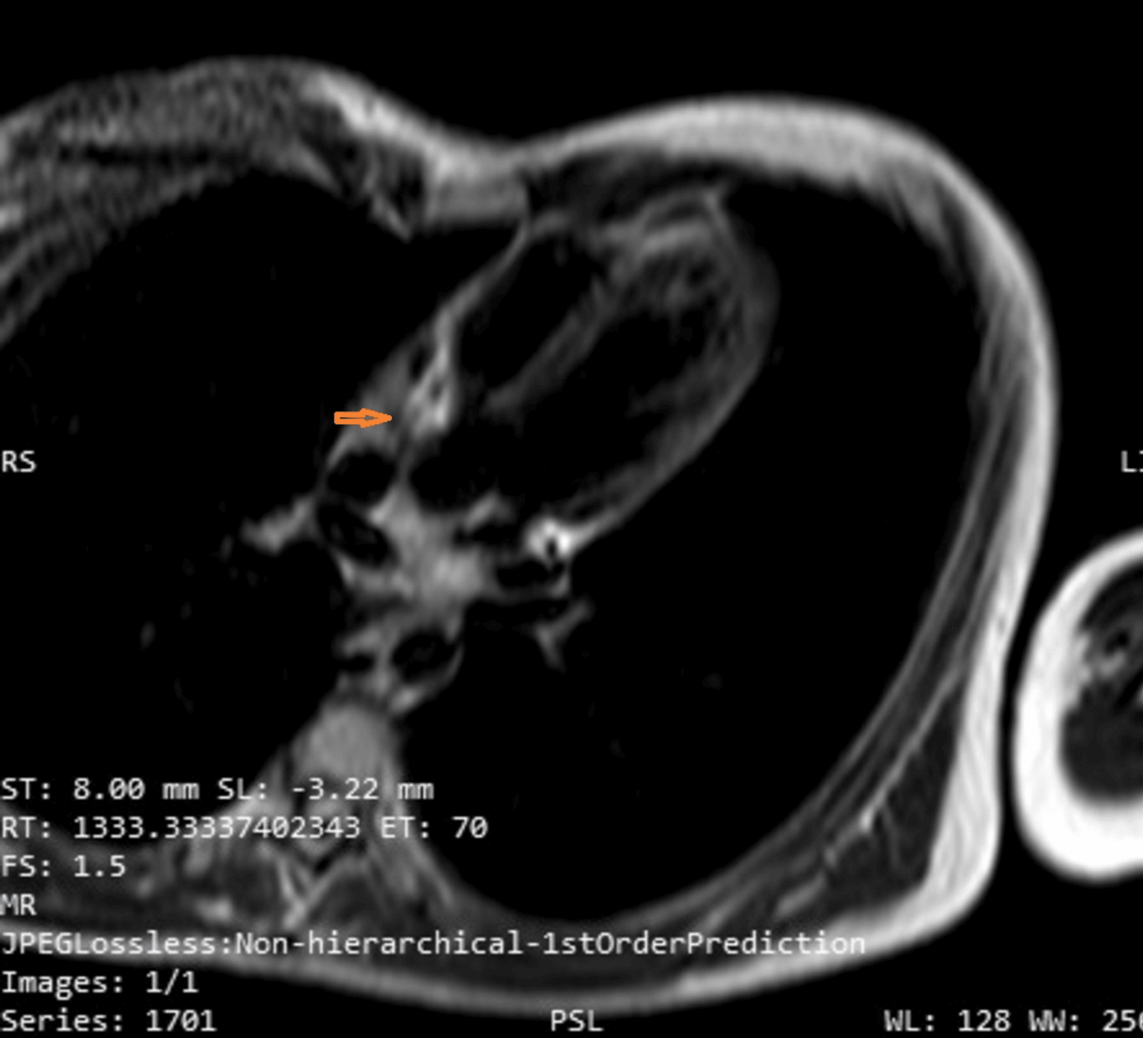
T2-weighted Turbo Spin Echo with Fat Saturation MRI sequence showing thickening of the right ventricular free wall with the muscular band extending from the tricuspid valve to the adjacent free wall (orange arrow).

Cardiac MRI was subsequently performed, which suggested evidence of hyper-trabeculation and thickening of the right ventricular free wall, with a possible muscular band extending from the tricuspid valve to the adjacent free wall. Additionally, the MRI indicated the involvement of the left ventricular myocardium, with basal and mid-cavity anteroinferior myocardial T2-weighted hyperintensity suggestive of myocardial edema. There was also a striae of post-contrast enhancement at the septal aspect of the RV basal septal myocardium, suggestive of infective or inflammatory pathology. This is depicted in Figures 5, 6.

A review of the literature was conducted based on MRI findings related to the inflammatory cardiac mass, and after conducting thorough laboratory investigations listed in Table 1, including blood cultures taken under infective endocarditis protocol to rule out infection, the patient was started on steroid therapy. She was prescribed prednisolone 30 mg once daily, tapered over a period of four weeks, along with metoprolol succinate 25 mg once daily.

**Table 1:** Lab investigation reports for the patient BNP: Brain natriuretic peptide; BUN: Blood urea nitrogen; CRP: C-reactive protein; ESR: Erythrocyte sedimentation rate

Investigation	Value	Reference values	Units
Hemoglobin (Hb)	13.2	(12-16)	g/dL
WBCs	6500 (Neutrophils 60.4%, Lymphocytes 34.1%)	(4000-10,000)	cells/microliter
Platelets	215,000	(150,000-400,000)	cells/microliter
BUN	10.8	(8.0-23.0)	mg/dL
Creatinine	0.78	(0.40-1.10)	mg/dL
NT-proBNP	Below 50	Below 50	pg/mL
Troponin I	Below 0.05	Below 0.05	ng/mL
ESR	14	0-20	mm for 1st hour
CRP	Negative		

**Figure 7:**
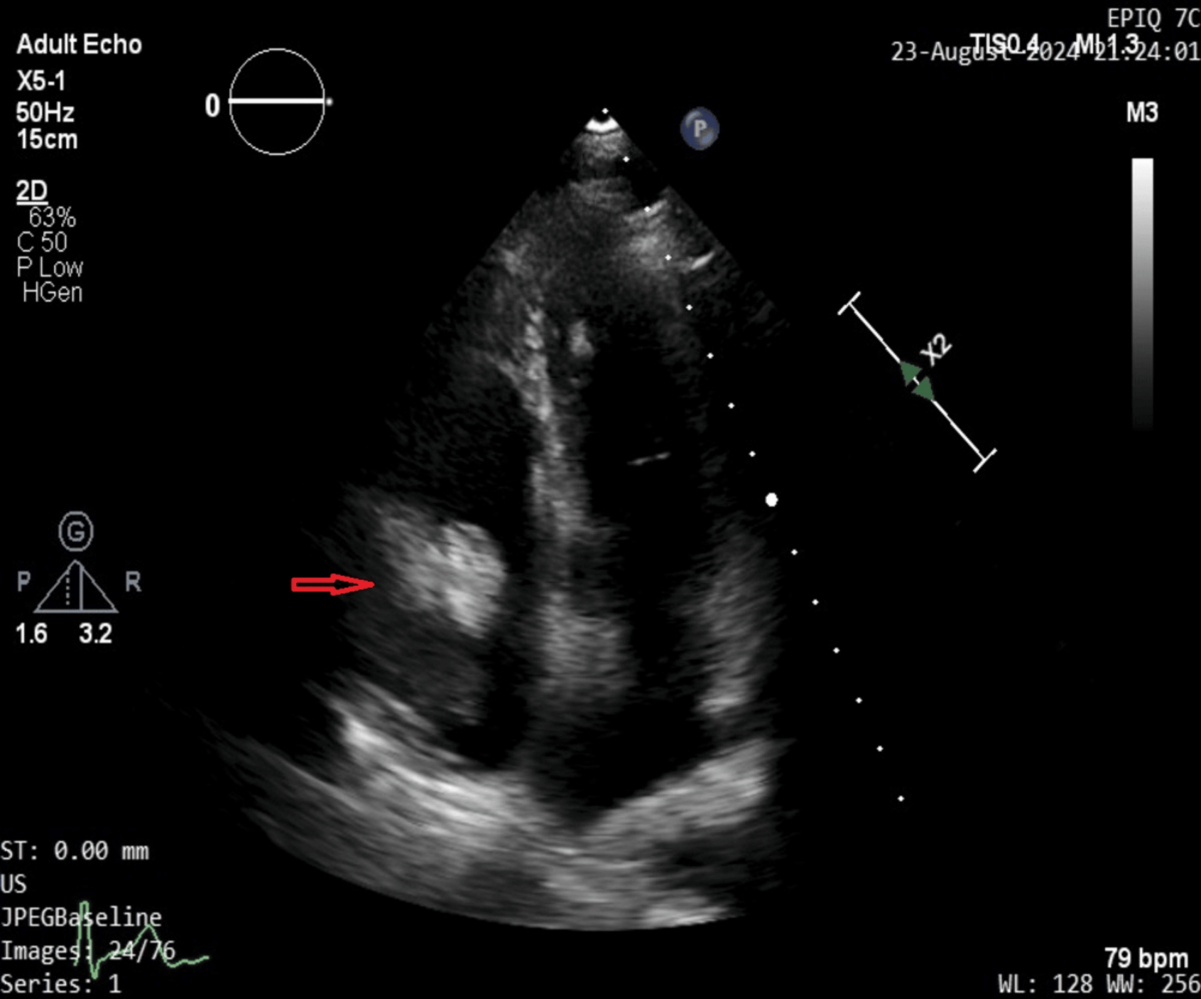
Transthoracic echocardiography at the end of four weeks suggesting persistence of the cardiac mass (red arrow) measuring 6 x 6 mm in the right atrium.

The symptoms improved partially over the treatment period, with her experiencing palpitations once every two or three weeks, lasting for a minute or so each time. It was not clear, however, whether the improvement can be attributed to steroids or due to our concomitant beta-blocker use. A repeat 2D Echo shown in Figure 7 however unexpectedly showed persistence of the cardiac mass at the end of treatment.

**Figure 8:**
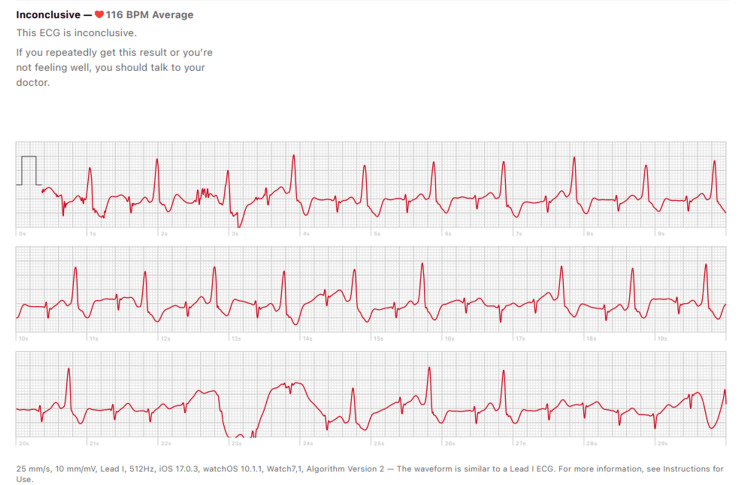
An Apple Smart Watch (Series 7) tracing from the patient revealing an episode of ventricular bigeminy.

Catheter-guided biopsy was considered as a potential diagnostic approach; however, it was ultimately not performed due to concerns about the risk of embolization and the difficulty in accurately targeting the mass with the catheter. The patient was continued on Tab metoprolol succinate 25 mg OD and her symptoms remained infrequent on follow-up. The clinical picture became more complex when a smartwatch tracing during the episodes of palpitations indicated the presence of ventricular premature contractions (VPCs) in a bigeminal pattern as shown in Figure 8. However, a 24-hour Holter monitor could not detect any VPCs, likely due to their infrequent occurrence.

## Discussion

The case of a 20-year-old female presenting with a right-sided cardiac mass located at the tricuspid valve highlights the diagnostic and therapeutic challenges associated with inflammatory cardiac masses in the young population. Initial symptoms of episodic palpitations prompted thorough evaluation, revealing a hyperechoic mass attached to the tricuspid valve via echocardiography, followed by cardiac MRI, which further characterized the mass and suggested inflammatory involvement of the myocardium.

Right-sided cardiac masses, such as thrombi, metastases, and primary tumors, are rare but often detected unexpectedly during transthoracic echocardiography (TTE). Thrombi are the most common intracavitary masses, while metastases are the most frequent neoplastic lesions in the heart. Primary cardiac tumors, although rare, are mostly benign, with myxomas being the most common type [6].

Despite initial symptom improvement during steroid therapy, the persistence of the cardiac mass at follow-up echocardiography raises critical considerations regarding the effectiveness of corticosteroid treatment for this particular case. While corticosteroids can effectively reduce inflammatory masses, as evidenced in various case studies, their efficacy can vary based on the underlying pathology and the specific characteristics of the mass itself. The lack of significant reduction in the mass size despite appropriate steroid therapy suggests that the inflammatory process may not have been adequately addressed, or that the mass may be of a different etiology that is inherently resistant to steroid treatment such as sarcoidosis or systemic lupus erythematosus (SLE), or neoplastic processes like cardiac myxoma or lymphoma. The detection of ventricular premature contractions (VPCs) in this case is significant as it may indicate underlying myocardial irritability, potentially linked to the inflammatory process associated with the cardiac mass.

Cardiac magnetic resonance (CMR) imaging has proven highly accurate in distinguishing between cardiac thrombi and tumors, and is useful for differentiating benign from malignant neoplasms [7]. It provides valuable information on tissue characteristics, vascularization, and contrast uptake, which helps differentiate these pathologies. Studies have shown its high accuracy in identifying thrombi, benign masses, and malignant masses [8]. The imaging findings, including myocardial edema and hyper-trabeculation, further complicate the clinical picture, indicating a multifaceted involvement of the heart that might require additional interventions, such as surgical evaluation. Surgical excision not only provides definitive tissue diagnosis through histopathological examination but can also relieve potential hemodynamic compromise posed by the mass [9,10]. Moreover, it may offer insight into whether the mass is indeed inflammatory, neoplastic, or related to other rare conditions such as cardiac sarcoidosis or autoimmune disorders, which may not be responsive to steroids. However, the presence of a muscular band instead of a distinct mass, as indicated by the MRI, along with the patient's young age and infrequent symptoms, raises uncertainty about the necessity of invasive management in this case [11].

Our case presents several unique features that differentiate it from other reported cases of inflammatory cardiac masses. Initial echocardiographic findings revealed a mass that was initially thought to be a tumor or thrombus which was refuted by the MRI findings thus reflecting common diagnostic challenges in distinguishing between these entities based solely on imaging. The presence of normal inflammatory markers, such as CRP and ESR, is atypical in cases of inflammatory cardiac masses, where one would generally expect elevated levels. Unlike other case reports that demonstrate the effectiveness of corticosteroids in reducing inflammatory cardiac masses, this case notably illustrates the persistence of the mass despite high-dose therapy [12]. This emphasizes the variability in response to treatment across different patients and the complexity involved in managing such cases. This discrepancy further complicates the clinical picture and underscores the need for a nuanced understanding of therapeutic response of these masses.

In future follow-ups, we will implement a regimen of serial echocardiographic assessments to monitor the size and morphological changes of the cardiac mass and longer continuous electrocardiographic rhythm monitoring to detect ventricular premature complexes. Continuous clinical evaluation will facilitate timely intervention should the mass demonstrate persistence or growth, potentially necessitating further imaging studies or a multidisciplinary approach to consider surgical intervention.

This case underscores the paucity in the available literature regarding the diagnosis and management of diseases presenting as cardiac masses and may stress the need for multidisciplinary approach in the management of cardiac masses, involving cardiologists, infectious disease specialists, and cardiac surgeons. A careful reevaluation of the mass through biopsy or surgical intervention may be warranted to establish a definitive diagnosis and guide further treatment options.

## Conclusions

In conclusion, this case of a young female with an inflammatory cardiac mass on the tricuspid valve demonstrates the challenges in diagnosing and managing rare cardiac lesions, particularly when corticosteroid therapy proves insufficient. The persistence of the mass highlights the need for alternative diagnostic strategies, including the careful consideration of biopsy or surgical excision in refractory cases. Additionally, advanced imaging techniques, such as cardiac MRI, proved invaluable in characterizing the mass and ruling out neoplastic or thrombotic etiologies. This case underscores the necessity of a multidisciplinary approach, tailored follow-up strategies, and further research to establish evidence-based guidelines for managing inflammatory cardiac masses in young patients.
